# Development of postbiotics by bioconverting whey using *Lactobacillus plantarum* SMFM2017-YK1 and *Limosilactobacillus fermentum* SMFM2017-NK1 to alleviate periodontitis

**DOI:** 10.1371/journal.pone.0263851

**Published:** 2022-10-06

**Authors:** Yuna Choi, Eunyoung Park, Yohan Yoon, Jimyeong Ha

**Affiliations:** 1 Department of Food and Nutrition, Sookmyung Women’s University, Seoul, Korea; 2 Risk Analysis Research Center, Sookmyung Women’s University, Seoul, Korea; Universidade Estadual de Ponta Grossa, BRAZIL

## Abstract

This study investigated the effects of whey bioconversion products (WBPs) produced by lactic acid bacteria on periodontal disease. WBPs were prepared by fermenting whey with seven lactic acid bacteria, *Limosilactobacillus fermentum* SMFM2017-CK1 (LF-CK1), *L*. *plantarum* SMFM2017-NK2 (LP-NK2), *Pediococcus pentosaceus* SMFM2017-NK1 (PP-NK1), *L*. *plantarum* SMFM2017-NK1 (LP-NK1), *L*. *paraplantarum* SMFM2017-YK1 (LPP-YK1), *L*. *plantarum* SMFM2017-YK1 (LP-YK1), and *L*. *fermentum* SMFM2017-NK1 (LF-NK1)]; the pH of the fermented whey was adjusted to 6.5, followed by centrifugation. WBPs were examined for their effect on cell viability and antimicrobial activity against periodontal pathogens. The selected WBPs were used in animal experiments. After inducing periodontitis through right mandibular first molar ligation, WBPs were administered orally for 8 weeks. After sacrifice, gene and protein expression analyses of genes related to inflammatory and oxidative stress were performed, and histopathological analysis of gingival tissue was conducted. Our results showed that LP-YK1 WBP (WBP produced by LP-YK1) and LF-NK1 WBP (WBP produced by LF-NK1) groups exerted higher anti-inflammatory and antioxidant effects compared to the control group (*p* < 0.05). Histopathological analysis revealed that infiltration of inflammatory cells and epithelial cell proliferation were reduced in the LP-YK1 WBP group. These results indicate that WBPs prepared with LP-YK1 can be used as a postbiotic to alleviate periodontitis.

## Introduction

Whey is a by-product of cheese production, and its production reaches > 160 million tonnes annually worldwide [1]. Whey is known to exert antibacterial and immune-enhancing effects and prevents cardiovascular disease and osteoporosis because of components such as β-lactoglobulin, *α*-lactalbumin, bovine serum albumin, lactoferrin, immunoglobulin, glycomacropeptides, and lactose [2, 3].

A recent study suggested that bioconversion of whey by lactic acid bacteria significantly reduced intracellular lipid accumulation and exhibited anti-adipogenic activity [4]. Another study [5] showed that when soy whey was fermented with *Lactobacillus plantarum* B1-6, the antioxidant activity of whey was elevated. In addition, a whey bioconversion product fermented by *Pediococcus pentosaceus* KI31 and *L*. *sakei* KI36 demonstrated anti-obesity activity [4]. These results indicate that even though lactic acid bacteria and whey have their own functional effects, when fermented together, their functionality is enhanced. However, till date, the effects of bioconversion products in alleviating periodontitis have not been examined.

Periodontitis is an inflammatory disease of the periodontal tissue caused by pathogenic bacteria [6]. Physical treatments of periodontal disease, such as scaling and root agglutination, fail to remove the pathogenic bacteria completely; thus, antibiotics are often used as alternate treatment options [7]. However, antibiotic use can result in antibiotic resistance, gastrointestinal disorders, and mucosal hypersensitivity.

Postbiotics are non-viable bacterial products or metabolic byproducts from probiotic microorganisms that have positive effects on the host or microbiota [8]. In addition, it was confirmed whether it could be used as postbiotics, functional bioactive compounds, generated in a matrix during fermentation [9].

Therefore, in this study, we developed bioconverted whey by fermentation using lactic acid bacteria to alleviate periodontitis, and it was confirmed whether this bioconverted whey can be used as postbiotics.

## Materials and methods

### Preparation of whey bioconversion products

Seven strains of lactic acid bacteria, *L*. *fermentum* SMFM2017-CK1 (LF-CK1), *L*. *plantarum* SMFM2017-NK2 (LP-NK2), *P*. *pentosaceus* SMFM2017-NK1 (PP-NK1), *L*. *plantarum* SMFM2017-NK1 (LP-NK1), *L*. *paraplantarum* SMFM2017-YK1 (LPP-YK1), *L*. *plantarum* SMFM2017-YK1 (LP-YK1), and *L*. *fermentum* SMFM2017-NK1 (LF-NK1), isolated from kimchi were used in this study. A loopful of each strain was inoculated in de Man, Rogosa, and Sharpe broth (MRS; Becton, Dickinson and Company, Franklin Lakes, NJ, USA) at 37°C for 24 h. Following incubation, 300 μL of each culture was inoculated into 30 mL whey solution (3 g whey powder with 30 mL distilled water, pasteurized at 80°C for 1 min), and incubated at 37°C for 24 h. During incubation, the pH decreased to 4.25 to 4.5, which was considered fermented. Each culture was adjusted to pH 6.5 using 1 N NaOH (Duksan Pure Chemical, Ansan, Korea) and centrifuged at 2,969 × *g* for 10 min. Subsequently, the supernatants were concentrated to 35% using a rotary evaporator (N-1200A, Eyela, Tokyo, Japan), and the concentrate was used as a whey bioconversion product (WBP).

### Evaluation of cytotoxicity

The effects of WBP on cell viability were measured using the 3-(4,5-dimethylthiazolyl-2)-2,5-diphenyltetrazolium bromide (MTT) assay, as described previously [10]. Briefly, human colon carcinoma HT-29 (KCLB30038) cells were cultured in Dulbecco’s Modified Eagle’s Medium (DMEM; Hyclone, Logan, UT, USA), supplemented with 10% fetal bovine serum (FBS; Hyclone) and 1% penicillin-streptomycin (PS; Gibco, Auckland, New Zealand), at 37°C in 5% CO_2_ for 48 h. HT-29 cells were trypsinized and transferred to 96-well plates at a concentration of 5 *×* 10^4^ cells/mL; each well contained 200 μL of medium. After incubation at 37°C in 5% CO_2_ for 24 h, the medium was replaced with fresh medium containing WBPs (20 μL WBP and 180 μL DMEM; each of the seven WBPs were added to separate wells) and cultured at 37°C in 5% CO_2_ for 24 h. Subsequently, 20 μL of colorimetric reagent MTT (0.5 g/mL) was added to each well with DMEM supplemented with 10% FBS, and incubated for 2–3 h. MTT is reduced to a purple formazan product by active mitochondrial reductase enzymes in viable cells; thus, the amount of formazan product is proportional to the number of viable cells [11]. The reaction was arrested by addition of 200 μL DMSO to each well, and formazan was quantified by measuring the optical absorbance at 540 nm using a microplate spectrometer (Take3, Epoch, BioTek, VT, USA). Cell viability was calculated using the following equation:

$$Cell\;viability=\frac{{OD}_{540nm}\;of\;the\;sample}{{OD}_{540nm}\;of\;the\;control}\times 100$$ (1)

The sample represented a group of cells treated with aliquots of 7 WBPs, and the control represented a group of cells treated with phosphate buffered saline (PBS; pH 7.4; 0.2 g of KH_2_PO_4_, 1.5 g of Na_2_HPO_4_·7H_2_O, 8.0 g of NaCl, and 0.2 g of KCl in 1 L of distilled water).

### Evaluation of antimicrobial activity

The antimicrobial activities of WBPs prepared with 7 strains of lactic acid bacteria against two periodontal pathogens (*Fusobacterium nucleatum* KCTC2640 isolated from cervico-facial lesion and *Aggregatibacter actinomycetemcomitans* KCTC3698 isolated from abscess) were analyzed. The antimicrobial activities of WBPs were investigated using the spot assay as described by Arnold et al. [12]. Two periodontal pathogens were stored in 1 mL vials (AES Chemunex, Combourg, France) at –70°C. One milliliter of each stock was then inoculated into 9 mL of Wilkins-Chalgren anaerobe broth (Oxoid, Hampshire, UK) and incubated at 37°C for 48 h in an anaerobic chamber (Coy Laboratory Products Inc., Grass Lake, MI, USA). One hundred microliters of periodontal pathogen inoculum was spread onto Columbia agar plates (BioMerieux, Marcy l’ Etoile, Lyon, France) using a spreader (SPL, Pocheon, Korea). Paper discs (Ø: 6 mm; Advantec Toyo Kaisha, Tokyo, Japan) containing 25 μL of WBPs were placed on the Columbia agar plates inoculated with pathogens. After incubation of the plates at 37°C under anaerobic conditions, the diameters (mm) of bacteria-free clear zones were measured.

### Animals

Thirty-two male Wistar rats (Orient Bio, Seongnam, Korea) weighing 180–220 g (6 weeks old) were housed under standard laboratory conditions (12 h light/dark cycle at 22–24°C). The animals were fed a solid diet and water *ad libitum*. The protocol for the animal study was approved by the Institutional Animal Care and Use Committee of Sookmyung Women’s University (SMWU-IACUC-1912-026).

### Induction of periodontitis and treatment

Before inducing periodontitis, rats were treated with antibiotics (1 mg/mL sulfamethoxazole and 200 μg/mL trimethoprim in water) by oral gavage for 2 days. To induce periodontitis, rats were anesthetized with an intraperitoneal injection of Zoletile (1 mg/mL) (Virbac, Carros, France) and rompun (Bayer, Berlin, Germany) [4:1 (v/v)]. A nylon thread ligature was placed around the right mandibular first molars of each rat. After 2 weeks, periodontitis-induced rats were treated with PBS (control, n = 8), WBP prepared with LP-YK1 (LP-YK1 WBP, n = 8), and WBP prepared with LF-NK1 (LF-NK1 WBP, n = 8); periodontitis-non-induced rats were treated with PBS (normal, n = 8). All treatments were orally administered daily for 8 weeks. The rats were then euthanized under general anesthesia with CO_2_ after 10 weeks.

### Transcriptome analysis

Total RNA was isolated from 30 μg of gingival tissue using a RNeasy mini kit (Qiagen, Hilden, Germany) following the manufacturer’s instructions. Complementary DNA (cDNA) was synthesized from total RNA using the Quantitect Reverse Transcription Kit (Qiagen) and amplified by quantitative polymerase chain reaction (qPCR). qPCR was performed with the Rotor-Gene SYBR Green PCR kit (Qiagen) using 1 μL of cDNA, 2.5 μL of forward and reverse primers, 6.5 μL of RNase-free water, and 12.5 μL of SYBR Green PCR Master Mix. Using Rotor-Gene Q (Qiagen), samples were incubated at 95°C for 5 min and amplified over 35 PCR cycles at 95°C for 5 s and at 60°C for 10 s. The primer sequences used for amplification are listed in Table 1. Cycle threshold (C_T_) values, determined by Rotor-Gene Q series software (Stratagene, La Jolla, CA, USA), were used to evaluate the relative expression of the target mRNA.

**Table 1:** Primer sequences of inflammatory and oxidative stress-related cytokines used for quantitative reverse transcription polymerase chain reaction (RT-qPCR).

Gene	Primer sequences (5’→3’)		Reference
*β-actin*	Forward	AAGTCCCTCACCCTCCCAAAAG	[13]
	Reverse	AAGCAATGCTGTCACCTTCCC	
*TNF-α*	Forward	AAATGGGCTCCCTCTCATCAGTTC	
	Reverse	TCTGCTTGGTGGTTTGCTACGAC	
*IL-1β*	Forward	CACCTCTCAAGCAGAGCACAG	
	Reverse	GGGTTCCATGGTGAAGTCAAC	
*IL-6*	Forward	TCCTACCCCAACTTCCAATGCTC	
	Reverse	TTGGATGGTCTTGGTCCTTAGCC	
*IL-10*	Forward	GCCCAGAAATCAAGGAGCATT	[14]
	Reverse	CAGCTGTATCCAGAGGGTCTTCA	
*SOD1*	Forward	CATTCCATCATTGGCCGTACT	
	Reverse	CCACCTTTGCCCAAGTCATC	
*GPx1*	Forward	GCGCTGGTCTCGTCCATT	
	Reverse	TGGTGAAACCGCCTTTCTTT	
*CAT*	Forward	GTACAGGCCGGCTCTCACA	
	Reverse	ACCCGTGCTTTACAGGTTAGCT	

### Protein analysis

Total protein was extracted from gingival tissue using PRO-PREP^TM^ protein extraction solution (Intron Biotechnology Inc., Seongnam, Korea), and protein concentration was estimated using the DC^TM^ Protein assay (Bio-Rad Laboratories, Hercules, CA, USA). Forty micrograms of total protein was resolved by SDS-PAGE, electrotransferred to a PVDF membrane, and probed with the appropriate primary and secondary antibodies in 5% skim-milk. Protein signals were visualized using ECL Select^TM^ (GE Healthcare Life Sciences, Marlborough, MA, USA). The primary antibodies, β-actin (SC-81178)(1:1,000), TNF-α (SC-52746)(1:300), IL-6 (SC-57315)(1:300), and secondary antibody [anti-mouse IgG HRP (SC 2005)(1:5,000)] were obtained from Santa Cruz Biotechnology (Dallas, TX, USA).

### Histopathologic analysis

After euthanasia, the lower right jawbone with ligature was removed. The tissue slices were fixed in 10% neutral-buffered formalin, embedded in paraffin, and sectioned. The sections were stained with hematoxylin and eosin (H&E). This analysis was performed at the Korea Pathology Technical Center (KPNT; Cheongju, Korea).

### Statistical analyses

Statistical analyses were performed using the general linear model of SAS^®^ (v.9.3, SAS Institute Inc., Cary, NC, USA). The least squares means among the treatments were compared using pairwise Student’s *t*-test at α = 0.05.

## Results and discussion

### Cytotoxicity of whey bioconversion products (WBPs)

**Fig 1:**
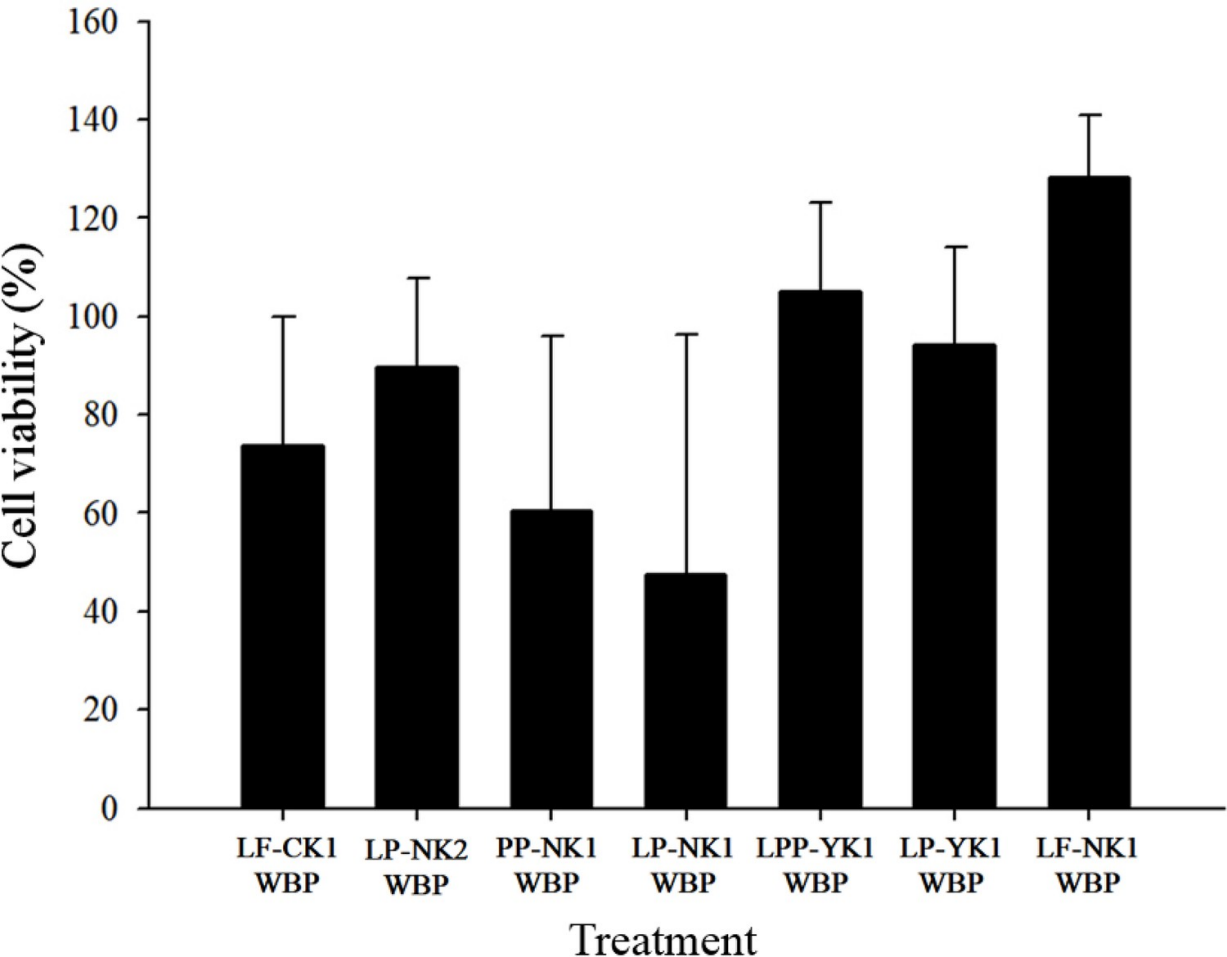
Viability of HT-29 cells treated with whey bioconversion products (WBPs) (OD_540_). LF-CK1 WBP: WBP prepared with *L*. *fermentum* SMFM2017-CK1, LP-NK2 WBP: WBP prepared with *L*. *plantarum* SMFM2017-NK2, PP-NK1 WBP: WBP prepared with *P*. *pentosaceus* SMFM2017-NK1, LP-NK1 WBP: WBP prepared with *L*. *plantarum* SMFM2017-NK1, LPP-YK1 WBP: WBP prepared with *L*. *paraplantarum* SMFM2017-YK1, LP-YK1 WBP: WBP prepared with *L*, *plantarum* SMFM2017-YK1, LF-NK1 WBP: WBP prepared with *L*. *fermentum* SMFM2017-NK1.

When HT-29 cells were treated with WBPs, such as LP-NK2 WBP, LPP-YK1 WBP, LP-YK1 WBP, and LF-NK1 WBP, the cell viabilities were > 90% (Fig 1), which is appropriate because the value was more than the 70% cut-off, as recommended by ISO 10993–5: 2009 (Biological Evaluation of Medical Devices part 5: Tests for *in vitro* Cytotoxicity), indicating that WBPs were biocompatible [15]. However, the cell viabilities of PP-NK1 WBP and LP-NK1 WBP were less than 60%. Such low cell viabilities might be caused by the excessive occurrence of the metabolites such as propionate and acetate produced in fermented milk [16]. This indicates that most of WBP may have no cytotoxic effects. Thus, WBPs can be applied to living organisms.

### Antimicrobial activity of WBPs

Most WBPs formed larger inhibition zones against *A*. *actinomycetemcomitans* KCTC3698 compared to those against *F*. *nucleatum* KCTC2640, and LP-YK1 WBP and LF-NK1 WBP formed relatively larger inhibition zones than the other WBPs (Table 2). According to a previous study [17], *A*. *actinomycetemcomitans* and *F*. *nucleatum* produce endogenous infections and are strongly associated with periodontitis. Therefore, it is important to inhibit *A*. *actinomycetemcomitans* and *F*. *nucleatum* to prevent and alleviate periodontitis. In this study, LP-YK1 WBP and LF-NK1 WBP showed significant antibacterial effects against *A*. *actinomycetemcomitans* and *F*. *nucleatum*. Thus, LP-YK1 WBP and LF-NK1 WBP treatments were further subjected to *in vivo* tests.

**Table 2:** Sizes (mean ± standard deviation; mm) of clear zones formed by seven whey bioconversion products against periodontal pathogens.

Group	Periodontal pathogen		Total Average
	*F*. *nucleatum*	*A*. *actinomycetemcomitans*	
	KCTC 2640	KCTC 3698	
LF-CK1 WBP	7.50±0.55	7.00±0.63	7.25±0.62
LP-NK2 WBP	6.83±0.75	6.00±0.00	6.42±0.67
PP-NK1 WBP	8.00±1.10	7.83±0.75	7.92±0.90
LP-NK1 WBP	8.00±0.89	7.17±0.75	7.58±0.90
LPP-YK1 WBP	6.17±2.56	8.67±1.03	7.42±2.27
LP-YK1 WBP	7.33±0.52	10.08±3.88	8.71±3.00
LF-NK1 WBP	6.83±0.41	9.33±1.75	8.08±1.78

LF-CK1 WBP: whey bioconversion product (WBP) prepared with *L*. *fermentum* SMFM2017-CK1, LP-NK2 WBP: WBP prepared with *L*. *plantarum* SMFM2017-NK2, PP-NK1 WBP: WBP prepared with *P*. *pentosaceus* SMFM2017-NK1, LP-NK1 WBP: WBP prepared with *L*. *plantarum* SMFM2017-NK1, LPP-YK1 WBP: WBP prepared with *L*. *paraplantarum* SMFM2017-YK1, LP-YK1 WBP: WBP prepared with *L*, *plantarum* SMFM2017-YK1, LF-NK1 WBP: WBP prepared with *L*. *fermentum* SMFM2017-NK1.

### Analysis of mRNA and proteins related to inflammation

**Fig 2:**
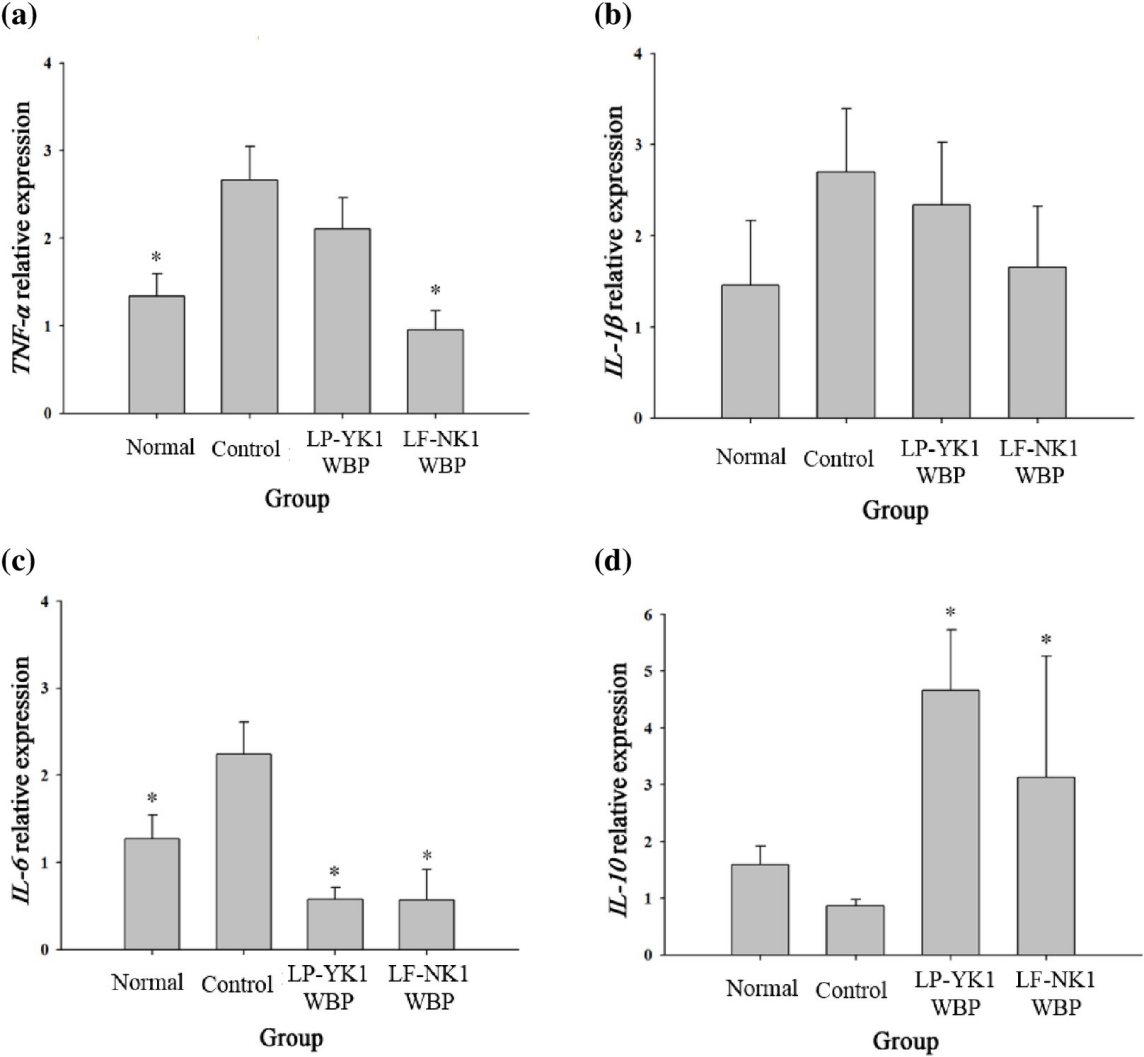
Relative expression of inflammatory cytokines in gingival tissue after treatment with fermented whey bioconversion products (WBPs) for 8 weeks. (a) *TNF-α* (b) *IL-1β* (c) *IL-6* (d) *IL-10*. Normal; Administration of phosphate buffered saline (PBS) without inducing periodontitis, Control; Administration of PBS after inducing periodontitis, LP-YK1 WBP; Administration of WBPs by *L*. *plantarum* SMFM2017-YK1 after inducing periodontitis, LF-NK1 WBP; Administration of WBPs by *L*. *fermentum* SMFM2017-NK1 after inducing periodontitis. *Statistically significant, compared to control group by pairwise Student’s *t*-test (*p* < 0.05).

**Fig 3:**
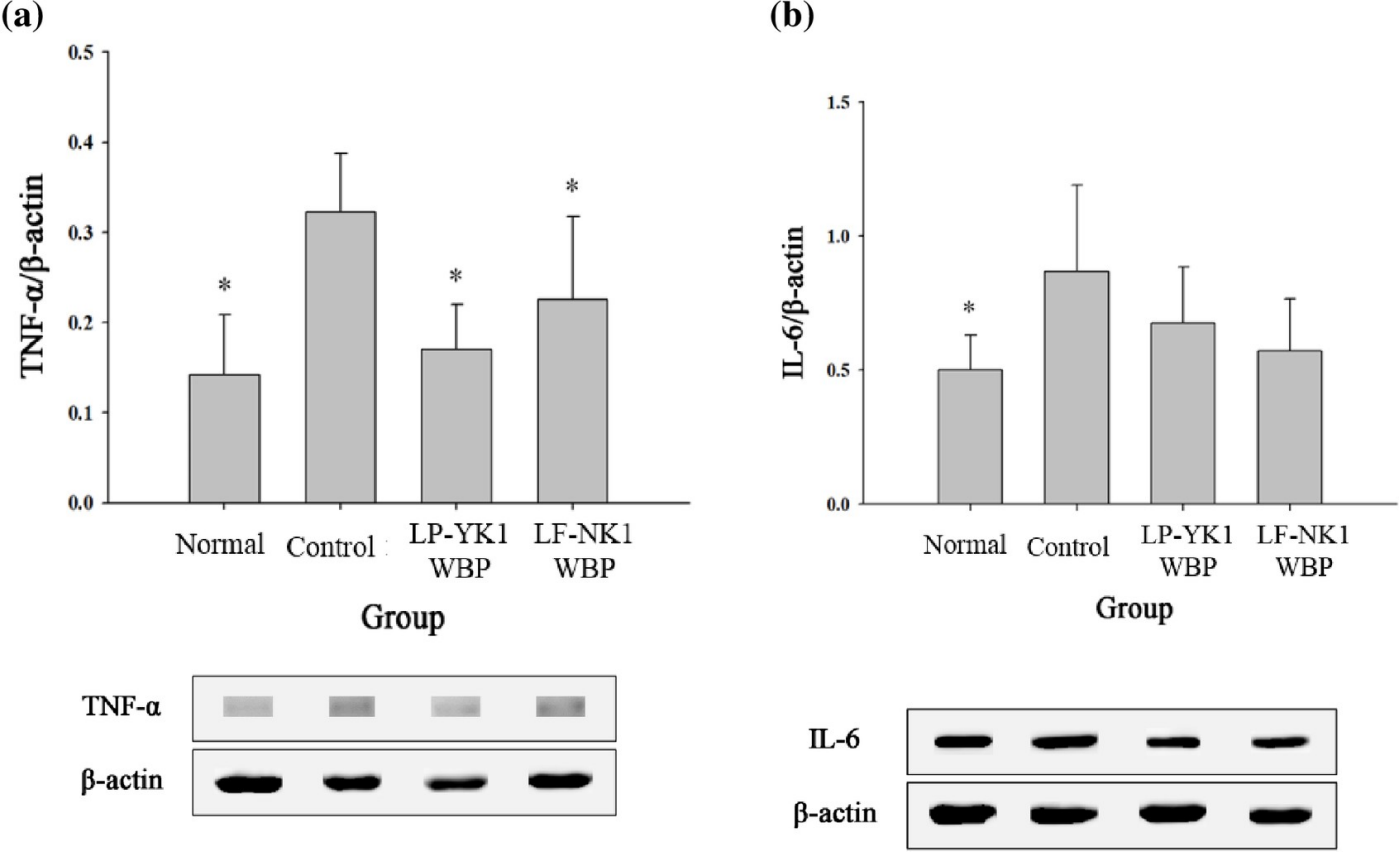
Analysis of protein levels of inflammatory cytokines in gingival tissue after treatment with fermented whey bioconversion products (WBPs) for 8 weeks. (a) TNF-α (b) IL-6. Normal: Administration of phosphate buffered saline (PBS) without inducing periodontitis, Control: Administration of PBS after inducing periodontitis, LP-YK1 WBP: Administration of WBPs by *L*. *plantarum* SMFM2017-YK1 after inducing periodontitis, LF-NK1 WBP: Administration of WBPs by *L*. *fermentum* SMFM2017-NK1 after inducing periodontitis. *Statistically significant, compared to control group by pairwise Student’s *t*-test (*p* < 0.05).

Analysis of gene expression levels in periodontal tissues revealed that *TNF-α*, a pro-inflammatory cytokine, exhibited the highest expression levels in the control group, whereas the expression levels were low in the normal, LP-YK1 WBP, and LF-NK1 WBP groups (*p* < 0.05) (Fig 2A). The protein expression levels of TNF-α were also lower (*p* < 0.05) in all groups (normal, LP-YK1 WBP, and LF-NK1 WBP) than in the control group (Fig 3A). TNF- α can regulate RANKL [18], the most determined cytokine involved in osteoclastogenesis, and is expressed higher in ligature-induced periodontitis models [19]. The expression levels of *IL-1β* and *IL-6*, which are known to be pro-inflammatory agents, were lower (*p* < 0.05) in LP-YK1 WBP and LF-NK1 WBP groups than in the control group (Fig 2B and 2C). The level of *IL-10*, an anti-inflammatory factor, was significantly higher (*p* < 0.05) in the LP-YK1 WBP and LF-NK1 WBP groups than in the control group (Fig 2D). As compared to those in the control group, the protein levels of IL-6 were generally lower in the normal group as well as in the LP-YK1 WBP and LF-NK1 WBP groups (Fig 3B). Obvious differences in the protein levels for IL-1β and IL-10 were not observed (data not shown). IL-6 is a signaling protein produced by several cell types, including macrophages and neutrophils, in response to stimuli such as infection, and its level is higher in patients with gingivitis and periodontitis than in healthy subjects [20]. In addition, in a recent study, bioconverted whey, postbiotics, were effective on inflammatory and antioxidant factors on periodontal tissue [21]. Also, another study showed that postbiotics had beneficial effects such as antibacterial, anti-inflammatory, and immune response control [22]. This suggests that LP-YK1 WBP and LF-NK1 WBP are effective in reducing inflammation in the periodontal tissue.

**Fig 4:**
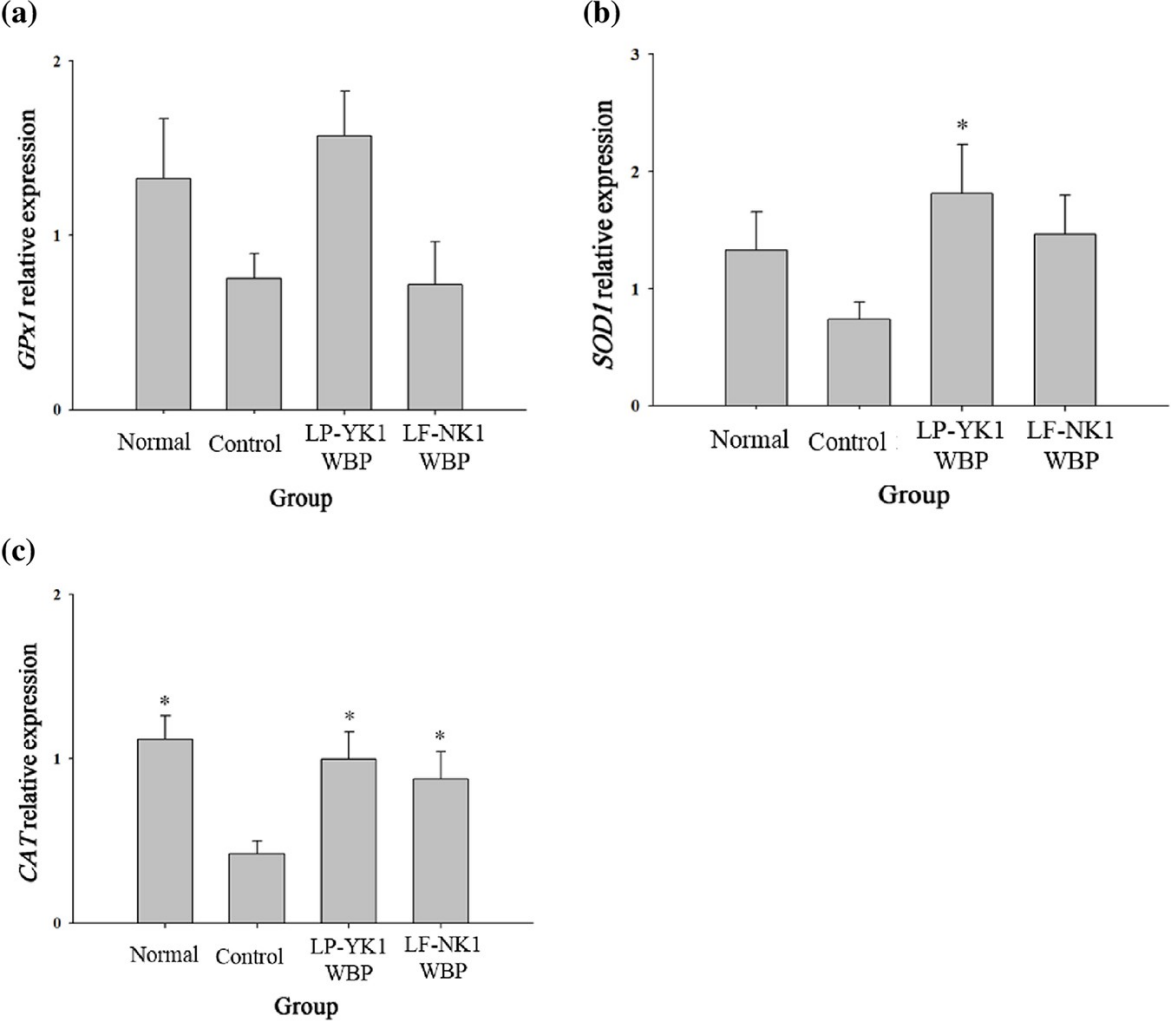
Relative expression of oxidative stress cytokines in gingival tissue after treatment of fermented whey bioconversion products (WBPs) for 8 weeks. (a) *Gpx1* (b) *Sod1* (c) *Cat*. Normal: Administration of phosphate buffered saline (PBS) without inducing periodontitis, Control: Administration of PBS after inducing periodontitis, LP-YK1 WBP: Administration of whey bioconversion product by *L*. *plantarum* SMFM2017-YK1 after inducing periodontitis, LF-NK1 WBP: Administration of whey bioconversion product by *L*. *fermentum* SMFM2017-NK1 after inducing periodontitis. *Statistically significant, compared to control group by pairwise Student’s *t*-test (*p* < 0.05).

Genes encoding known antioxidant enzymes, *Gpx-1*, *Sod1*, and *Cat* showed higher expression levels in the LP-YK1 WBP and LF-NK1 WBP groups than those in the control group (Fig 4). Several studies suggested that decreased activities of antioxidants such as SOD, CAT, and GPx were associated with periodontitis [23]. When immune responses are initiated by harmful oral bacteria, reactive oxygen species (ROS) are produced in periodontal tissue which can act as toxic substances that cause cell damage when overproduced. In addition, periodontitis patients have been found to exhibit oxidative stress, with increased lipid peroxidation and ROS levels [24]. In the present study, treatment with LP-YK1 WBP and LF-NK1 WBP increased the expression of antioxidant genes, and thus, the generation of ROS in periodontal tissues might be decreased. Therefore, fermented WBPs administered for 8 weeks after inducing periodontitis were considered to be effective in alleviating oxidative stress in periodontal tissues.

### Manifestation in tissue

**Fig 5:**
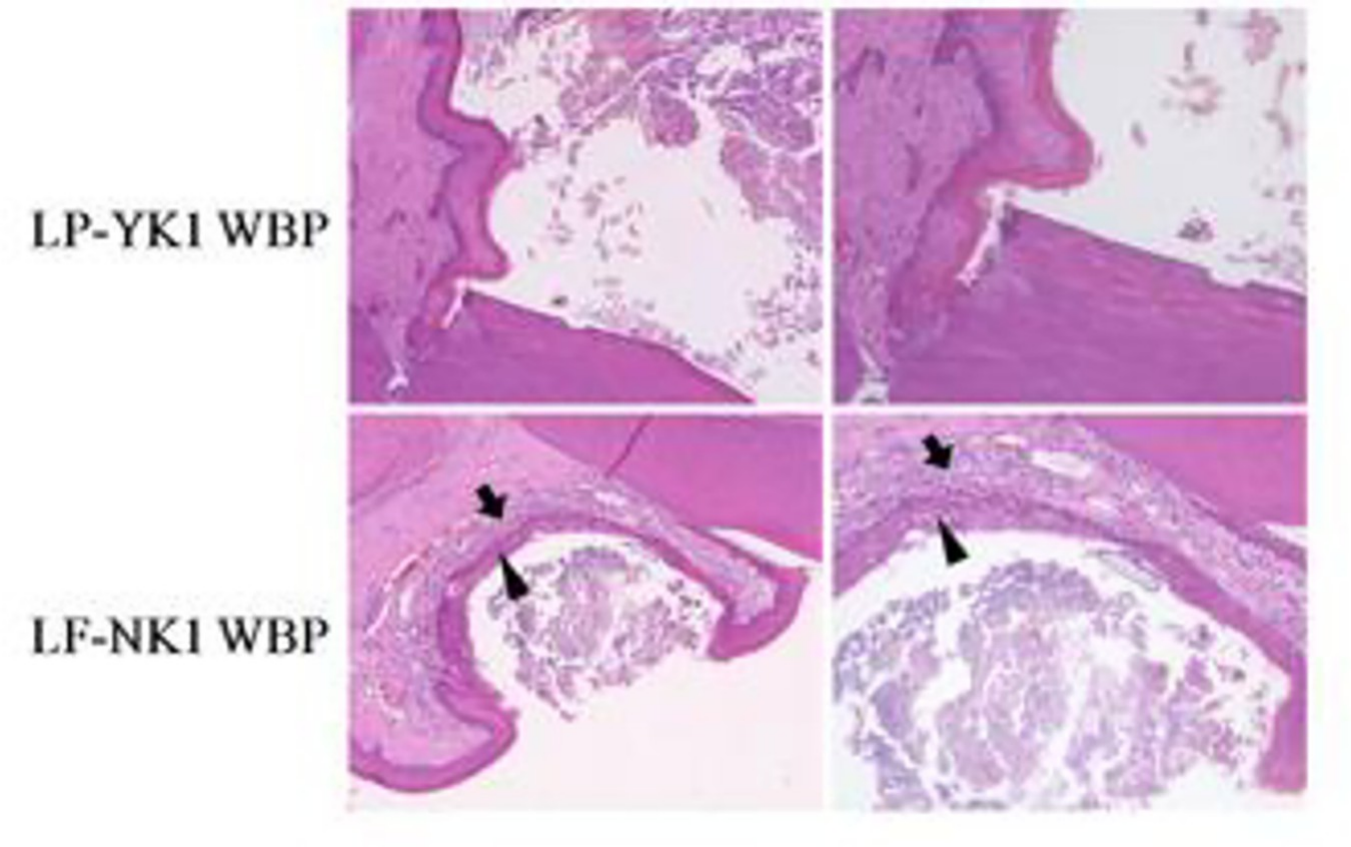
Histopathologic analysis of mandibular first molar tooth regions from rats administered with whey bioconversion products (WBPs) and their effects on periodontitis. LP-YK1 WBP: Administration of WBPs produced by *L*. *plantarum* SMFM2017-YK1 after inducing periodontitis, LF-NK1 WBP: Administration of WBPs by *L*. *fermentum* SMFM2017-NK1 after inducing periodontitis. Arrow: inflammatory cell infiltration, Head of arrow: epidermal hyperplasia.

Periodontitis through ligation causes the infiltration of inflammatory cells and epithelial proliferation. Histopathological analysis of animal periodontal tissue did not indicate the presence of inflammation in rats administered LP-YK1 WBP; however, LF-NK1 WBP did not alleviate inflammatory cell infiltration and epithelial cell proliferation (Fig 5). This indicates that the application of LP-YK1 WBP reduces infiltration by inflammatory cells and epithelial cell proliferation in periodontitis tissue. LP-YK1 WBP may contain antimicrobial materials to *A*. *actinomycetemcomitans* and *F*. *nucleatum*, but LF-NK1 WBP may contain less antimicrobial materials than LP-YK1 WBP, which was inferred by the data in Table 2.

## Conclusions

LP-YK1 WBP produced by LP-YK1 and LF-NK1 WBP produced by LF-NK1 were not cytotoxic and showed antibacterial effects against *F*. *nucleatum* and *A*. *actinomycetemcomitans*. When these WBPs were administered to periodontitis-induced rats for 8 weeks, the protein and mRNA levels of pro-inflammatory agents decreased, while the levels of those with anti-inflammatory and antioxidant activities increased. In addition, a low level of inflammation was observed in the periodontal tissues of the group treated with LP-YK1 WBP. Periodontitis is mainly caused by pathogenic bacteria, and LP-YK1 WBP is not toxic to cells and exhibits antimircobial effects on major pathogenic bacteria F. nucleatum and A. actinomycetemcomitans, reducing infectious factors and increasing antioxidant efficacy to reduce periodontitis. Therefore, the LP-YK1 WBP developed in this study can be used as a postbiotic to alleviate periodontitis.
